# Geographic Clustering of Leishmaniasis in Northeastern Brazil^1^

**DOI:** 10.3201/eid1506.080406

**Published:** 2009-06

**Authors:** Albert Schriefer, Luiz H. Guimarães, Paulo R.L. Machado, Marcus Lessa, Hélio A. Lessa, Ednaldo Lago, Guilherme Ritt, Aristóteles Góes-Neto, Ana L.F. Schriefer, Lee W. Riley, Edgar M. Carvalho

**Affiliations:** Universidade Federal da Bahia, Salvador, Brazil (A. Schriefer, L.H. Guimarães, P.R.L. Machado, M. Lessa, H.A. Lessa, E. Lago, G. Ritt, A.L.F. Schriefer, E.M. Carvalho); Universidade Estadual de Feira de Santana, Feira de Santana, Brazil (A. Góes-Neto); University of California School of Public Health, Berkeley, California, USA (L.W. Riley)

**Keywords:** Leishmania braziliensis, leishmaniasis, American tegumentary leishmaniasis, disease distribution, disseminated leishmaniasis, geographic clustering, Brazil, parasites, research

## Abstract

To determine whether disease outcomes and clades of *Leishmania braziliensis* genotypes are associated, we studied geographic clustering of clades and most severe disease outcomes for leishmaniasis during 1999–2003 in Corte de Pedra in northeastern Brazil. Highly significant differences were observed in distribution of mucosal leishmaniasis versus disseminated leishmaniasis (DL) (p<0.0001). Concordance was observed between distribution of these disease forms and clades of *L. braziliensis* genotypes shown to be associated with these disease forms. We also detected spread of DL over this region and an inverse correlation between frequency of recent DL diagnoses and distance to a previous DL case. These findings indicate that leishmaniasis outcomes are distributed differently within transmission foci and show that DL is rapidly spreading in northeastern Brazil.

## Abstract 2

Different forms of this disease are spreading rapidly in distinct geographic clusters in this region.

Leishmaniasis accounts for ≈2 million disability-adjusted life years in ≈90 countries, most of which are in the developing world (*1*). The past 3 decades have witnessed accumulation of much knowledge about the host-parasite relationship, especially about host immune responses against *Leishmania* spp. The focus on immunity reflects in part the central role played by the immune system for pathogenesis of leishmaniasis (*2*,*3*) and the need for appropriate prophylaxis against this heterogeneous group of diseases that remain uncontrolled and are increasing in prevalence and incidence (*4*,*5*). Therefore, better understanding and control of this disease demand additional approaches, especially investigations that focus on the parasite, the host environment, and their relationship to clinical outcomes.

Differences in geographic distribution of distinct clinical forms of American tegumentary leishmaniasis (ATL) have long been recognized in Andean countries in South America. To a large extent, this phenomenon seems to be determined by the prevalence of various *Leishmania* spp. in diverse environments. For example, in Ecuador and Peru, the highlands harbor almost exclusively localized cutaneous leishmaniasis (CL) cases caused by several *Leishmania* spp., whereas mucosal leishmaniasis (ML) is mostly limited to the Amazon rain forest and caused by *L*. *braziliensis* (*6*,*7*). Conversely, observations such as those in the Peruvian lowlands, where *L*. *braziliensis* causes CL throughout the country but ML is almost exclusively found in Amazonian provinces (*7*), lend support to the hypothesis that strain variability within a species may influence the form and distribution of ATL. To understand whether geographic segregation of ATL outcomes occurs within a more confined geographic space (foci of ATL transmission), we compared how cases of ML and disseminated leishmaniasis (DL) were distributed during 1999–2003 in Corte de Pedra in northestern Brazil, where active transmission of parasites from a complex population of *L*. *braziliensis* to humans occurs.

## Materials and Methods

### Study Area

Corte de Pedra is composed of 20 municipalities in a rural area previously dominated by the Atlantic rain forest. *Lutzomyia* (*Nyssomyia*) *whitmany* and *Lu*. (*Nyssomyia*) *intermedia* sandflies that transmit *L. braziliensis* are endemic in the local fauna. This biome had not undergone any major changes during the period of the study. Residents in this area work mostly in agriculture, often in primary or secondary forests. There is little population migration in or out of this region. Study participants’ mean time of residence at their addresses at the time of diagnosis and parasite sampling was 17 years; >90% of the study participants lived on farms.

### Disease Definitions

CL was defined as a disease with <10 ulcerative skin lesions without evidence of mucosal involvement. DL was defined as a disease with >10 nodular, acneiform, or ulcerative lesions spread over the skin of >2 body areas. ML was defined as a disease with metastatic mucosal lesions affecting the nose, palate, pharynx, or larynx and not contiguous with primary cutaneous lesions. Patients who simultaneously satisfied the definitions for ML and DL were classified as patients with DL showing mucosal involvement (MDL). This classification distinguishes these patients from those with classic ML, which usually shows skin involvement compatible with CL. All patients had their diagnosis confirmed by detection of parasites in culture aspirates or by histopathologic analysis, and a delayed-type hypersensitivity reaction.

### Patients with ATL

For geographic comparisons of disease distribution, participants with ATL were classified according to disease definitions into 3 groups: 30 patients with ML, 30 with DL, and 17 with MDL. Diagnoses were made during 1999–2003 in Corte de Pedra. Geographic coordinates of residence sites of these ATL patients were obtained by using a Brunton Multi-Navigator global positioning system apparatus (Brunton Company, Riverton, WY, USA), which has a range precision of 15 m. To characterize dynamics of DL spread within Corte de Pedra, we mapped the residences of 66 patients with DL with or without mucosal involvement. These patients received a diagnosis during 1993–2002 and represented ≈50% of all DL and MDL patients who came to the health post in Corte de Pedra during that period.

We analyzed clinical records of 102 patients with DL and 6,297 patients with ATL in the health post during 1993–2003. We also used geographic coordinates for another group of 21 patients (9 with *L*. *braziliensis* clade C isolates and 12 with clade A plus D isolates) whose isolated parasites had been used to define clades (i.e., subpopulations) of *L*. *braziliensis* genotypes circulating in Corte de Pedra, as determined by random amplified polymorphic DNA analysis (*8*). All ATL case-patients in this study were self-referred and diagnosed in 1 health post that treats ≈70% of patients with leishmaniasis in the region.

### Geographic Distribution of Patients with ATL

High-resolution distribution of ATL cases was determined by acquisition of geographic coordinates of likely places of disease transmission by a global positioning system. Because leishmaniasis is believed to be transmitted mostly within plantations, where residents of the region live and work, patient residences were used as reference points for standardization purposes. Collected data were statistically compared as described below and plotted for visual inspection onto a high-definition satellite photograph of Corte de Pedra (ENGESAT, Curitiba, Brazil) by using ArcInfo version 8.3 software (Environmental Systems Research Institute Inc., Redlands, CA, USA).

### Statistical Analyses

We studied distribution of ML and DL in Corte de Pedra by dividing the area into inner and coastal regions and compared frequencies of each of these forms of ATL in these 2 regions by using the χ^2^ test. We also confirmed how patients with ML, DL, and MDL clustered by using the Cuzick and Edwards test in the geostatistical package Clusterseer version 2.2.4 (Terraseer Inc., Ann Arbor, MI, USA), which is sensitive for detection of geographic patterns. Times that patients resided at given places of residence were compared using Kruskal-Wallis 1-way analysis of variance. To analyze whether proximity to a DL patient was accompanied by an increased frequency of DL diagnosis among dwellers of the region, we used a geographic information system (ArcInfo version 8.3 software) to measure distances between the residence of each new patient during 1998–2002 (recent cases) and residences of all patients in the preceding 12 months (past cases). Resulting data were stratified into discrete distance intervals of 0–2,500, 2,501–5,000, 5,001–7,500, 7,501–10,000, and 10,001–12,500 m from patients with recent cases. Linear regression analysis was then used to compare the number of past cases to distances from patients with recent cases. A p value <0.05 was considered significant.

## Results

**Figure 1:**
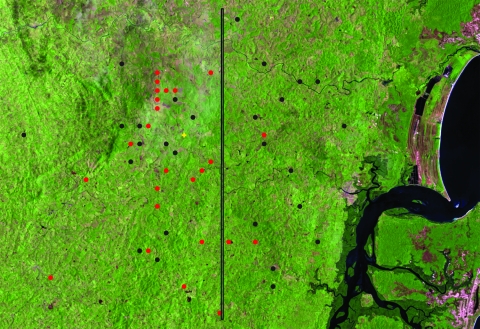
Satellite view of distribution of patients with disseminated leishmaniasis (DL; black circles) and patients with mucosal leishmaniasis (ML; red circles) in Corte de Pedra, Brazil, 1999–2003. Vertical line divides the region into inner (left) and coastal (right) areas of similar size. Total number of patients shown is smaller than the number of corresponding patients because of overlap of geographic coordinates for some patients. For details, see Materials and Methods. p = 0.00005, for data analyzed by using the Cuzick and Edwards test in Clusterseer version 2.2.4 (Terraseer Inc., Ann Arbor, MI, USA). The yellow mark indicates the health post.

A satellite view of Corte de Pedra with an arbitrary line dividing it into inner and coastal regions of approximately equal areas is shown in Figure 1. A total of 15 patients with DL were distributed in the coastal region and 15 patients with DL were distributed in the inner region (p>0.05, by χ^2^ test). Patients with ML were rarely observed in the coastal area during the study period; 87% of patients with ML were observed in the inner region (p<0.01, by χ^2^ test). To confirm the differences observed, we compared distribution of patients with ML and those with DL by using the Cuzick and Edwards test, which directly compares 2 sets of geographic events. Results were highly significant (p = 0.00005), which indicated that these 2 types of leishmaniasis spread differently throughout Corte de Pedra.

Because we had detected different subpopulations (clades) of *L*. *braziliensis* genotypes defined by random amplified polymorphic DNA analysis in this area (*8*), we also determined whether distributions of patients with ML and those with DL overlapped distributions of some of those clades. Distributions of patients with ML and those with DL overlapped exclusively clades C and A plus D, respectively (p>0.05, by Cuzick and Edwards test). All other comparisons showed significant differences in distributions (p<0.03). Thus, different types of ATL caused by the same parasite species are distributed differently, even within a specific ATL-endemic region. Overlap between specific *L*. *braziliensis* subpopulations and patients with ML or DL also suggests that this phenomenon may be influenced by distribution of parasite genotypes in the region. However, this proposal needs to be tested by using a method that is capable of accurately identifying genotypes for a large panel of isolated parasites.

**Figure 2:**
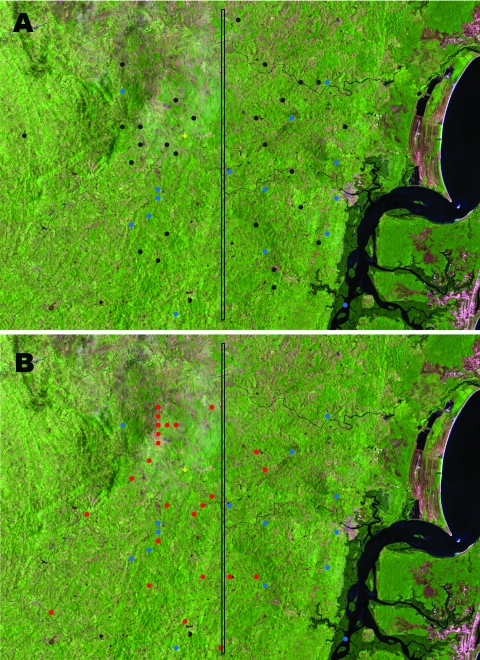
Satellite view of distribution of patients with disseminated leishmaniasis (DL) limited to the skin, patients with mucosal leishmaniasis (ML), and patients with DL showing mucosal involvement (MDL) in Corte de Pedra, Brazil, 1999–2003. A) Black circles indicate patients with DL, and blue circles indicate patients with MDL. B) Red circles indicate patients with ML, and blue circles indicate patients with MDL. Vertical line divides the region into inner (left) and coastal (right) areas of similar size. Total number of patients shown is smaller than the number of corresponding patients because of overlap of geographic coordinates for some patients. For details, see Materials and Methods. p = 0.8 in panel A and p = 0.00003 in panel B for data analyzed by using the Cuzick and Edwards test in Clusterseer version 2.2.4 (Terraseer Inc., Ann Arbor, MI, USA). The yellow mark indicates the health post.

Observation of higher frequencies of ML in the inner region of Corte de Pedra suggested that extrinsic local factors not related to subpopulations of parasites might influence disease outcome. Because <40% of patients with DL also have mucosal involvement, we addressed this issue by comparing the distribution of patients with MDL with those with DL or ML. Patients with MDL showed a distribution pattern similar to that of patients with DL in this region (p = 0.8, by Cuzick and Edwards test; Figure 2) but different from that of patients with ML (p = 0.00003).

**Figure 3:**
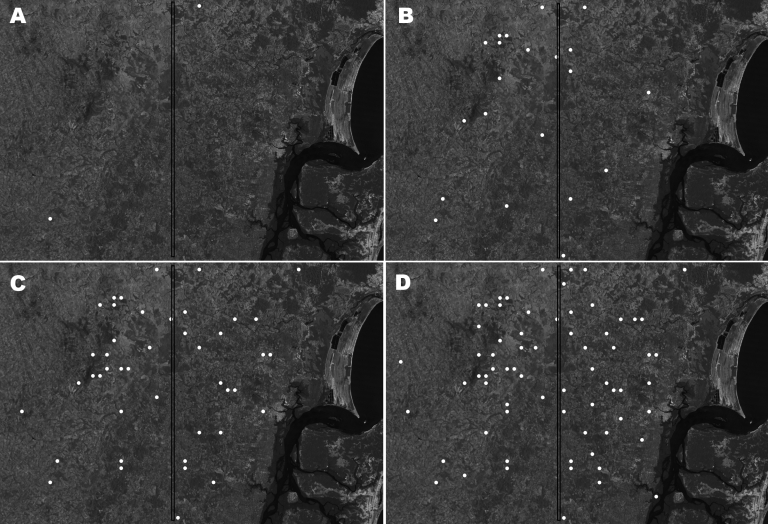
Satellite view of progressive spread of disseminated leishmaniasis in Corte de Pedra, Brazil, 1993–2002. Cumulative distributions of cases within affected areas are indicated by white circles. A) 1993, B) 1993–1996, C) 1993–1999, D) 1993–2002. The vertical line divides the region into inner (left) and coastal (right) areas of similar size.

**Figure 4:**
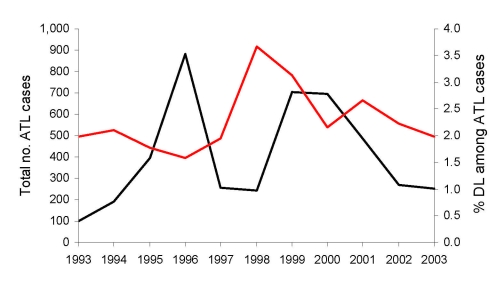
Distribution of American tegumentary leishmaniasis (ATL) (red line) and incidence of disseminated leishmaniasis/total ATL cases (black line) in Corte de Pedra, Brazil, 1993–2003.

**Figure 5:**
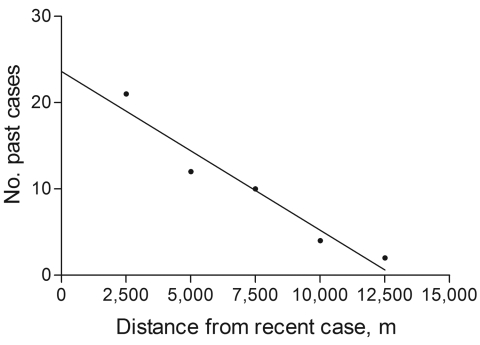
Linear regression comparing number of cases of disseminated leishmaniasis (past cases) diagnosed in the 12 months preceding a newly diagnosed case of DL (recent case) and distance to these recent cases, in increments of 2,500 m, in Corte de Pedra, Brazil, 1993–2003. p = 0.0061, r^2^ = 0.94.

The broader distribution of DL compared with ML was surprising because only during the past decade has disseminated disease become more frequently diagnosed in Corte de Pedra. To better understand the dynamics of the spread of DL, we mapped the distribution of this disease in this region during 1993–2002. Our results show that up to 1996, DL, similar to ML, was concentrated mostly in the inner region of Corte de Pedra (Figure 3). However, the next 6 years showed progressive spread of DL to the coastal region until it reached an even distribution over the entire area (Figure 3). Two peaks in the incidence of DL paralleled the spread of DL in both regions of Corte de Pedra in the past decade (Figure 4). An increase was observed in the incidence of DL, with progressive spread into the inner region up until 1996, and another increase resulted in spread of this disease into the coastal region during 1998–2001. These 2 increases and the general increasing trend in incidence (Figure 4) also indicate that DL occurs in a pattern distinct from those of ML and CL. Linear regression (Figure 5) showed a significant inverse correlation between distance from a newly diagnosed case and frequency of diagnosis of DL patients in the preceding 12 months. These findings reinforce our previous suggestion that DL is an emerging disease (*9*) and that this form of leishmaniasis spreads in part through multiple outbreaks.

## Discussion

A distinct geographic clustering of disease forms secondary to *L*. *braziliensis* infection was found in the study region. The overlap of ML and DL with genotypes of parasites associated with such outcomes in this area (clades C and A plus B, respectively) (*8*) suggests a cause–effect relationship. However, small sample sizes used to represent subpopulations of *L*. *braziliensis* genotypes found in Corte de Pedra and the low significance level obtained in comparisons prompt further studies before we can conclude whether the intraspecies parasite polymorphism affects disease distribution over an affected area. Our findings suggest a complex organization of the types of ATL within foci of active disease transmission.

The combination of human population movement in the study region and the usual long latency period of ML suggest cautious interpretation of our data. For ML, months to years may elapse between parasite acquisition and development of mucosal lesions. Thus, residences of patients with ML at the time of diagnosis may not reflect actual distributions of patients at the time of infection with the parasites. However, the likelihood of this possibility was precluded because participants’ mean time of residence at their addresses at the time of ATL diagnosis was 17 years; mean times did not differ between the study groups.

Use of patients’ homes as primary sources of geographic coordinates was another limitation intrinsic to the retrospective study design. A more accurate approach would be to delineate the personal activity spaces of each patient with ML or DL and then perform the geographic analyses. However, estimation of personal activity spaces is based on recollection of data gathered by questionnaires administered to the study participants regarding past information such as places of residence, history of migrations, work-related activities, and sites for these activities. Such information would be more reliable in a prospective study. Nonetheless, taking into account that residents of Corte de Pedra are mostly engaged in agriculture, often conducted within walking distance from their homes, and that population migration within this region is limited, we expect collection of geographic coordinates based on place of residence to be a fair approximation of actual places of infection with *Leishmania* spp. in most cases. More specifically, in our sample, >90% of patients had lived on farms for more than a decade.

Wide geographic differences in distribution of types of ATL have been reported for Ecuador and Peru (*6*,*7*). Our findings extend this observation to a smaller geographic setting that involves foci of endemic parasite transmission to humans. Distinct distributions of ML and DL over an area of only 10,000 km^2^ support the complexity of *L*. *braziliensis* reported for this region (*8*). We hypothesize that some subpopulations of the parasite may be associated with disease manifestations and with factors that affect the transmission dynamics of *L*. *braziliensis* strains, such as various sandfly vectors present in the study area (*9*,*10*). However, other nonhuman hosts and reservoirs may also play a role.

African trypanosomiasis and schistosomiasis can illustrate the effects of parasite and vector heterogeneities on geographic distribution of these diseases. In eastern and southern Africa, infections with *Trypanosoma brucei rhodesiense* are characterized by an acute form of sleeping sickness. In western and central Africa, infections with *T*. *brucei gambiense* are characterized by a chronic form of this disease (*11*,*12*). Regions in which *Schistosoma hematobium* and *S*. *mansoni* are endemic are affected by the presence of snails of the genera *Biomphalaria* and *Bulinus*, respectively, in infested bodies of water. Although *S*. *hematobium*, which causes urinary schistosomiasis, was the predominant species in Egypt up to the 1930s (*13*), *S*. *mansoni*, which causes hepatointestinal disease, has progressively replaced *S*. *hematobium* in the Nile Delta and more recently in Upper Egypt (*14*). This change was paralleled by the concomitant replacement of *Bulinus truncatus* snails by *Biomphalaria alexandrina* snails in the affected areas, largely caused by human intervention and modification of the ecology for irrigation purposes (*15*–*17*).

Our findings also show that DL, which is a novel severe and difficult-to-treat form of leishmaniasis (*18*), is rapidly emerging and spreading within Brazil, 1 of the 5 countries with 90% of human cases of tegumentary leishmaniasis worldwide (*1*). The unique pattern of DL incidence (Figure 4), which shows 2 peaks, indicates that this form of leishmaniasis may occur as outbreaks. Conversely, the increased frequency of DL in persons living near persons with recent cases of this disease may be caused by other factors, such as uneven human population distribution and vector densities in the area and other potential environmental factors that affect parasite reservoirs. Human-to-vector-to-human transmission of parasites may also play a role in this form of American leishmaniasis. However, anthroponotic cycles are not considered to be predominant in ATL, except for a few reports suggesting that this mode of transmission may occur with *L*. *chagasi* within large urban areas in northeastern Brazil (*19*–*21*).

The 2 increases in the incidence of DL preceded similar increases in the total number of ATL cases by ≈2 years. One possible explanation would be that factors affecting transmission of DL respond faster to changes in environmental parameters than those of CL and ML. The roles of climate and ecologic changes in leishmaniasis in regions near Corte de Pedra have been reported by Franke et al. (*22*). These authors reported a significant correlation between the southern oscillation of the El Niño phenomenon and the incidence of visceral leishmaniasis in the state of Bahia, Brazil. The major increase in the incidence of visceral leishmaniasis detected in that study occurred during 1995–1996, a period coincident with the first peak of DL shown in Figure 4. Although mechanisms responsible for the phenomena we describe remain elusive, we believe that information on clustering of disease types, increased frequency of DL among persons living near persons with recently diagnosed cases of the same disease, and predictive behavior of the incidence of DL relative to that of ATL may be used for better management and control of ATL.
